# Crystallization of gas-selective nanoporous graphene by competitive etching and growth: a modeling study

**DOI:** 10.1038/s41598-019-41645-9

**Published:** 2019-03-26

**Authors:** Soumajit Dutta, Mohammad Tohidi Vahdat, Mojtaba Rezaei, Kumar Varoon Agrawal

**Affiliations:** École Polytechnique Fédérale de Lausanne (EPFL), Rue de l’Industrie 17, Sion, 1951 Switzerland

## Abstract

A robust synthesis methodology for crystallizing nanoporous single-layer graphene hosting a high density of size-selective nanopores is urgently needed to realize the true potential of two-dimensional membranes for gas separation. Currently, there are no controllable etching techniques for single-layer graphene that are self-limiting, and that can generate size-selective nanopores at a high pore-density. In this work, we simulate a unique chemical vapor deposition based crystallization of graphene on Cu(111), in the presence of an etchant, to generate a high density (>10^13^ cm^−2^) of sub-nanometer-sized, elongated nanopores in graphene. An equilibrium between the growth rate and the etching rate is obtained, and beyond a critical time, the total number of the carbon atoms and the edge carbon atoms do not change. Using an optimal first-order etching chemistry, a log-mean pore-size of 5.0 ± 1.7 (number of missing carbon atoms), and a pore-density of 3 × 10^13^ cm^−2^ was achieved. A high throughput calculation route for estimating gas selectivity from ensembles of thousands of nanopores was developed. The optimized result yielded H_2_/CO_2_, H_2_/N_2_ and H_2_/CH_4_ selectivities larger than 200, attributing to elongated pores generated by the competitive etching and growth. The approach of competitive etching during the crystal growth is quite generic and can be applied to a number of two-dimensional materials.

## Introduction

Nanometer-thick nanoporous membranes have received much attention owing to their potential to yield large molecular flux^1–5^. Especially, the development of nanoporous single-layer graphene films has been intensively pursued for energy-efficient separations^6–11^. Single-layer graphene is atom-thick, is chemically and thermally stable in a non-oxidative environment, is stable in air at least up to 200 °C^12^, and is one of the strongest materials known (elastic modulus of 1 TPa)^13^. However, the synthesis of nanoporous graphene lattice with a narrow pore-size-distribution (PSD) and a high pore-density is not trivial^14^. Since the pristine graphene lattice is impermeable, one needs to controllably etch the lattice to generate gas selective nanopores. The lattice etching needs to be carried out in a controlled manner to open only those pores that are selective to the gas molecule of interest, and to avoid large non-selective pores. Here, selectivity could be size-selectivity (molecular sieving)^15^, shape-selectivity (entropic selectivity)^16^, and adsorption-selectivity^17^. Among these, size-selectivity has the potential to yield the highest separation selectivity, where a complete blockage of larger gas molecules can be achieved.

Recently, we demonstrated that low-density of intrinsic defects in graphene can yield an attractive separation performance^18^. H_2_ permeance greater than 10^−7^ mol m^−2^ s^−1^ Pa^−1^ was achieved from a pore-density of 10^10^cm^−2^, while the membrane yielded H_2_/CH_4_ selectivities in the range of 6–25. More recently, hydrogen sieving from hydrocarbons (CH_4_ and C_3_H_8_) using plasma- and ozone-based etching of single-layer graphene was achieved with H_2_ permeance reaching 2 × 10^−6^ mol m^−2^ s^−1^ Pa^−1^ and with H_2_/CH_4_ and H_2_/C_3_H_8_ selectivities exceeding 30 and 200, respectively^19^. Competitive diffusion rather than competitive adsorption determined gas selectivity with H_2_/C_3_H_8_ mixture separation factor exceeding the corresponding ideal selectivity. Interestingly, molecular simulations and transport calculations have shown that nanoporous graphene lattice, hosting size-selective nanopores at a high enough pore-density, exhibits ultrahigh gas permeance^15–17,20–24^. For instance, with a pore-density of 10^12^cm^−2^, a H_2_ permeance larger than 10^−5^ mol m^−2^ s^−1^ Pa^−1^ (close to 1 million gas permeation units, GPU) has been predicted by several molecular dynamics (MD) simulations^15,17,22^. Jiang *et al*. calculated energy barriers of 0.22 and 1.60 eV for H_2_ and CH_4_, respectively, from a hydrogen-functionalized pore-10 (pore made by etching 10 atoms) using the density functional theory (DFT)^15^. Their study employed a high pore-density of 5.3 × 10^13^ cm^−2^. The low energy barrier for H_2_ (0.22 eV) combined with the high pore-density corresponds to a H_2_ permeance of 10^−5^ mol m^−2^ s^−1^ Pa^−1^ (assuming activated transport with a pre-exponential coefficient of 10^11^ s^−1^). Using classical molecular dynamics (MD) simulations for the translocation of H_2_ across an unfunctionalized pore-10, Sun *et al*. estimated a H_2_ permeance of 10^−3^ mol m^−2^ s^−1^ Pa^−1^, and a H_2_/N_2_ and H_2_/CH_4_ selectivities greater than 10 and 100, respectively^22^. Therefore, the presence of small nanopores (close to pore-10) in graphene at a high pore-density can enable both high selectivity and permeance.

**Figure 1:**
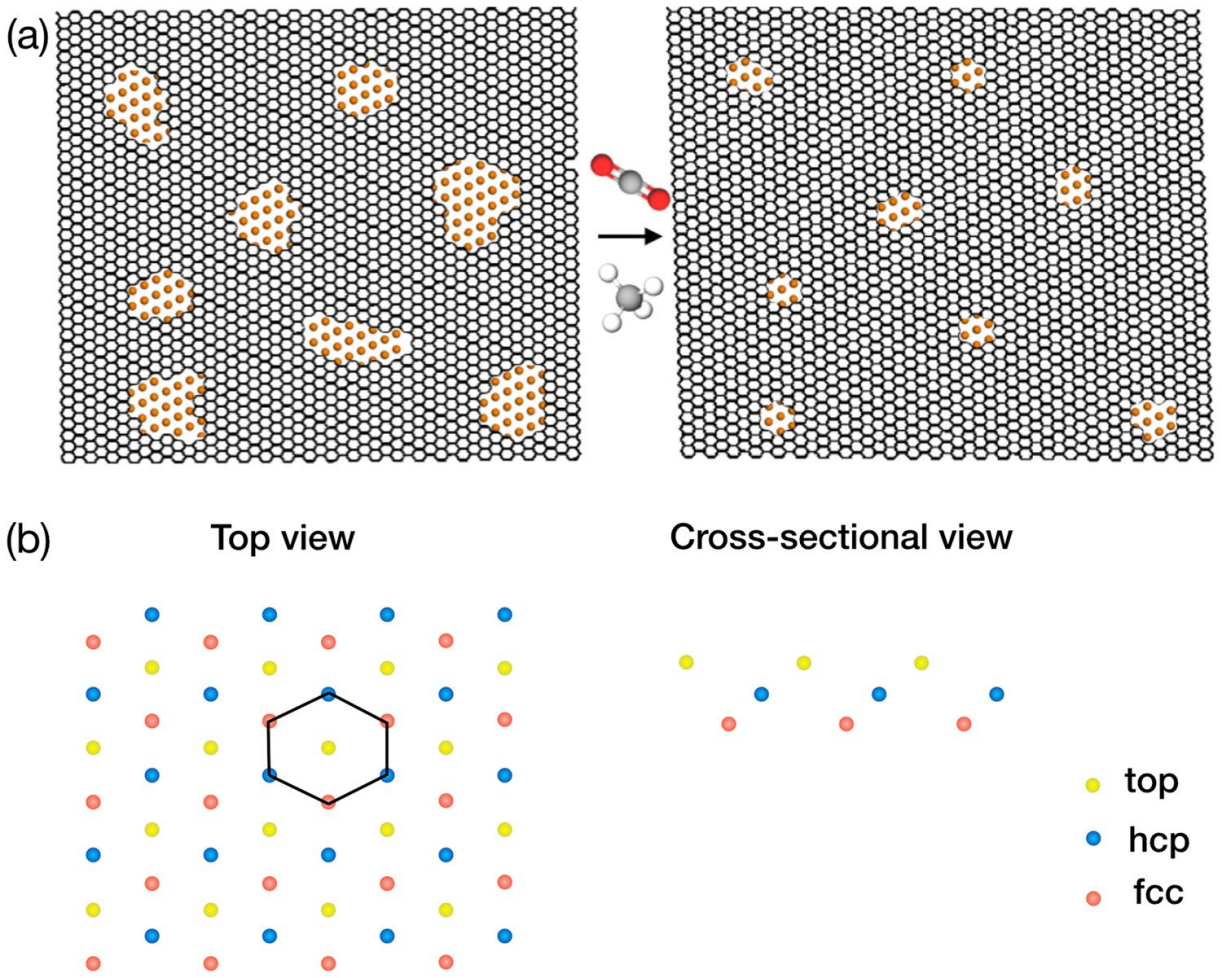
(**a**) An illustration of the method introduced here to generate nanoporous graphene with a high-density of size-selective nanopores using a competitive growth and etching during the CVD of graphene. (**b**) Representation of the Cu(111) lattice showing top three atomic layer of Cu. The hexagonal ring of graphene is shown as a result of deposition of carbon atoms selectively on the hcp and fcc sites.

The conventional pore etching techniques (UV/ozone, plasma, ion-bombardment, ion drilling) suffer from a tradeoff between the PSD and the pore-density, especially in the sub-nanometer pore regime, attributing to a much higher reactivity of the pore edges compared to that of the basal plane^25,26^. Therefore, one of the biggest bottlenecks in the realization of graphene membranes is the synthesis of nanoporous graphene possessing size-selective nanopores at a high pore-density. Herein, using a kinetic Monte Carlo (kMC) algorithm, we report a promising crystallization methodology capable of incorporating a high-density of size-selective, elongated nanopores in graphene in a scalable manner. This is achieved by subjecting a nanoporous graphene lattice (typically obtained by conventional etching methods) to the chemical vapor deposition (CVD) conditions in the presence of an etchant (Fig. 1a). The interplay between the competitive etching and the growth modulates the PSD while maintaining a high pore-density. Graphene growth was simulated on the Cu(111) surface using the established kMC protocols involving a high-temperature CH_4_ dehydrogenation kinetics^27^, reported ab-initio calculations on the diffusivity of reaction intermediates^28^, and the nucleation events of the carbon radical on Cu(111) surface. For etching, a first-order etching chemistry with etching rate proportional to the number of nanopore edge atoms was applied. A high-throughput transport calculation from the ensemble of nanopores indicates that attractive separation selectivities (H_2_/CO_2_, H_2_/CH_4_ and H_2_/N_2_ > 50) at a high pore-density (>10^13^ cm^−2^) is indeed possible by such approach.

## Results

### Generation of graphene lattice on Cu by kMC

For the simulation of graphene growth on Cu(111), a Cu surface of the dimension 26.5 nm × 26.5 nm was created which contained 12480 Cu atoms. In this fashion, three atomic layers of Cu were created (Fig. 1b). As per the calculation of Riikonen *et al*., the sites on the Cu surface corresponding to the underlying hcp and the fcc cites are the most probable sites for the adsorption of carbon atoms^29^. Accordingly, all deposition and surface diffusion events were restricted to the hcp and fcc sites. This routes makes sure that pristine graphene can be formed on the Cu surface (Fig. 1b). As carbon atoms can only be adsorbed on two of the three sites, each carbon can have maximum of three nearest neighbors and six next to next neighbors. To ensure that the chosen Cu area was representative of a larger surface, a periodic boundary condition was applied.

**Figure 2:**
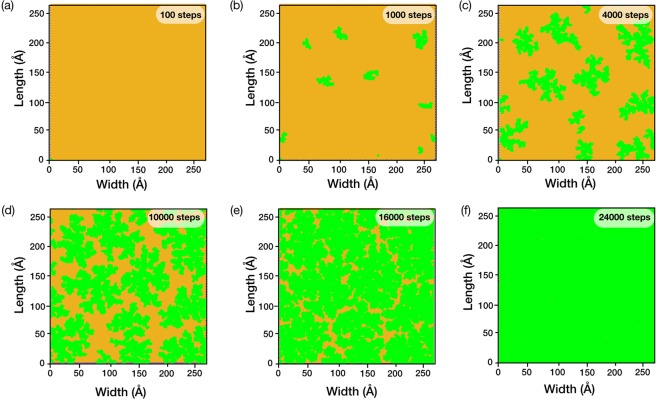
Graphene growth by CVD of CH_4_ on Cu(111) as a function of the number of CH_4_ deposition steps. (**a**) 100 steps. (**b**) 1000 steps. (**c**) 4000 steps. (**d**) 10000 steps. (**e**) 16000 steps. (**f**) 24000 steps. The orange and the green domains represent Cu and graphene, respectively.

The kMC algorithm used CH_4_ as a growth precursor (partial pressure of 45 Pa, representing the low-pressure CVD, LPCVD) at 1000 °C on the bare Cu(111) surface (kMC parameters in Table 1). The subsequent carbon deposition led to dendritic 10-nm-sized graphene grains (Fig. 2). Dendritic grains have been experimentally observed by Shin *et al*. under a hydrogen excluded CVD environment^30^, confirming that the applied algorithm correctly predicts the crystallization of graphene in these conditions. The dendritic structure can be explained by the rapid grain growth rate in comparison to the diffusion of C precursor. In the absence of H_2_, the activation energy for breaking a single carbon-carbon bond is much higher (1.3 eV) than the activation energy for the free diffusion of the precursors (0.5 eV)^28^. Therefore, once the bond is formed, there is a low probability of a bond cleavage.

**Table 1:** kMC parameters.

kMC Parameters	Values	Reference
Pressure, P	45 Pa	
Temperature, T	1273 K	
Area, A	26.5 × 26.5 nm^2^	
Dehydrogenation of CH_4_, ${E}_{act,d,C{H}_{4}(s)}$	1.77 eV	^27^
Dehydrogenation of CH_3_, ${E}_{act,d,C{H}_{3}(s)}$	1.53 eV	^27^
Dehydrogenation of CH_2_, ${E}_{act,d,C{H}_{2}(s)}$	1.13 eV	^27^
Dehydrogenation of CH, *E*_*act*,*d*,*CH*(*s*)_	1.97 eV	^27^
Bond dissociation energy with next neighbor carbon, *E*_*N*_	1.3 eV	^28^
Bond dissociation energy with next to next neighbor carbon, *E*_*NN*_	0.6 eV	^28^

The nucleation density of graphene domains was ca. 2 for an area of 100 nm^2^ (Fig. 2b). We note that the nucleation density in our simulation was much larger than experimental findings (typically, 1 per 100 nm^2^ to 1 per inch^2^, depending upon the CH_4_ concentration and annealing history of Cu). The high nucleation density in this study is attributed to a high CH_4_ deposition rate implemented in this study primarily to reduce the computational time. The grains grew progressively as a function of CH_4_ deposition steps (Fig. 2b–e). A complete coverage of the Cu surface was obtained in 24000 deposition steps (Fig. 2f). Since the grains in this study were aligned attributing to the periodic arrangement of the hcp and fcc sites of Cu, they could merge seamlessly without any grain-boundary defects (Fig. 2f).

### Evolution of nanoporous graphene

**Figure 3:**
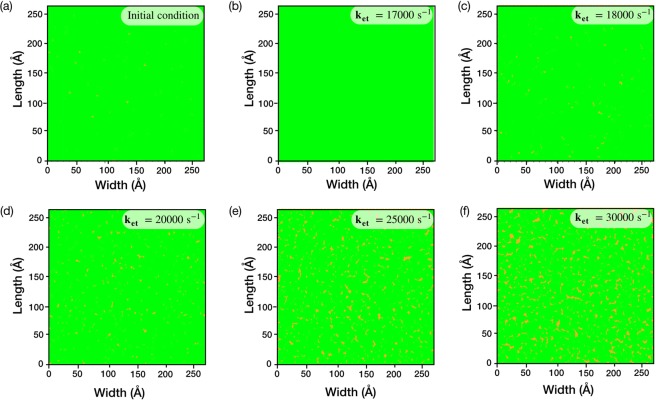
The generation of nanoporous graphene by simultaneous growth and etching in the CVD conditions. (**a**) Initial nanoporous structure of graphene before starting the kMC algorithm. The evolution of graphene with an etching rate constant of (**b**) 17000 s^−1^, (**c**) 18000 s^−1^, (**d**) 20000 s^−1^, (**e**) 25000 s^−1^ and (**f**) 30000 s^−1^.

**Figure 4:**
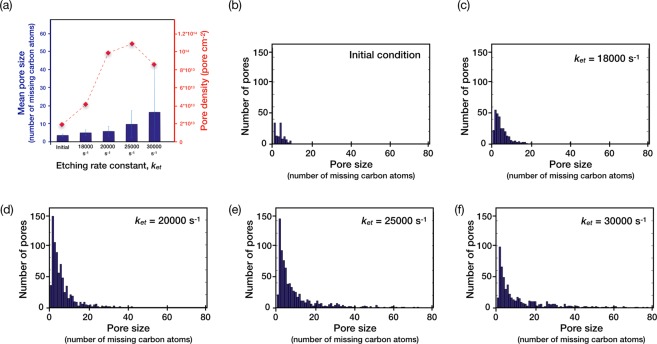
The porosity of graphene after CVD growth in the presence of an etchant. (**a**) Mean pore-size and pore-density as a function of the etching rate constant. The error bar in the mean pore-size corresponds to the standard deviation in the PSD. (**b**–**f**) Resulting PSD of the nanoporous graphene lattice as a function of the etching rate constant. PSD for the initial condition (**b**) and nanoporous graphene generated using the kMC algorithm with etching rate constants of (**c**) 18000 s^−1^, (**d**) 20000 s^−1^, (**e**) 25000 s^−1^, and (**f**) 30000 s^−1^.

To generate size-selective nanopores at a high pore-density, herein, we introduce the concept of a competitive growth and etching of the nanoporous graphene lattice. For this, a pristine single-layer graphene lattice without any defects was generated on the surface of Cu(111). Then, the lattice was decorated with defects (Fig. 3a) with a mean pore-size corresponding to missing 3.6 ± 0.8 carbon atoms and with a pore-density of 1.8 × 10^13^ cm^−2^ (Fig. 4a,b). Experimentally, such pore-density and PSD can be achieved by exposing CVD graphene to ion-bombardment^9^. Finally, the kMC algorithm was applied to study the evolution of these defects (PSD and mean pore-size) when the lattice was subjected to the competitive growth and etching. Keeping the growth rate was fixed, the etching rate was varied. Etching was ensued using a hypothetical etchant following a first-order etching chemistry with respect to the number of carbon edges. A limitation on etching chemistry was imposed such that the etchant could only etch graphene lattice from the pore-edge terminations, while the basal plane etching was not allowed. This is true for mild etchants such as carbon dioxide in the CVD conditions (Fig. S1)^31^. The etching rate constants (17000 to 30000 s^−1^) were chosen such that the etching rates were close to that of the growth rate. In experiments, the etching rates can be varied by varying the partial pressure of the etchant gas. Applying the kMC algorithm led to nanoporous structures with the PSD and the mean pore-size dependent upon the etching rate constant (Figs. 3 and 4).

The etching rate constant of 17000 s^−1^ led to a lattice resembling the pristine graphene lattice, indicating that the nanopores shrank completely (Fig. 3b). This implies that at this etching rate constant, the growth rate was higher compared to the etching rate. Rate constants higher than 17000 s^−1^ did not shrink the nanopores. In all of these cases, a log-normal PSD with a mean pore-size higher than the initial pore-size was obtained. In terms of missing carbon atoms, the mean pore-size increased from 3.6 ± 0.8 to 5.0 ± 1.7 (18000 s^−1^), 5.8 ± 2.7 (20000 s^−1^), 9.7 ± 7.5 (25000 s^−1^), and 16.4 ± 24.8 (30000 s^−1^). Extremely high pore-densities were obtained, reaching up to 10^14^ pores cm^−2^ (Fig. 4a).

**Figure 5:**
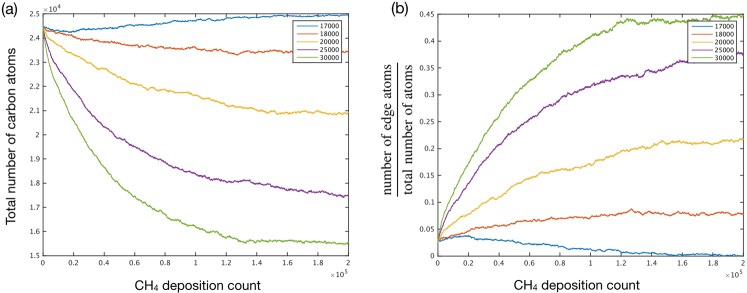
The evolution of (**a**) the number of all carbon atoms of graphene, and (**b**) the ratio of number of edge atoms with the total number of atoms as a function of the deposition count.

To understand the temporal evolution of nanoporous graphene, the total number of carbon atoms in the graphene lattice as a function of time was probed (Fig. 5a). In all cases, the total number of carbon atoms changed rapidly initially and become constant after a period of time. The number of carbon atoms increased at the etching rate constant of 17000 s^−1^, consistent with our previous observation. On the other hand, the number of carbon atoms decreased at the higher etching rate constants. At the rate constant of 18000 s^−1^, the number of carbon atoms reduced by 4.1% (from 2.45 × 10^4^ to 2.35 × 10^4^). The number of carbon atoms reduced by 14.7, 28.6 and 36.7% at the etching rate constants of 20000, 25000 and 30000 s^−1^, respectively. Accordingly, the pore-density increased to 4.2 × 10^13^, 9.9 × 10^13^, 10.9 × 10^13^, and 8.4 × 10^13^ cm^−2^ at the etching rate constants of 18000, 20000, 25000 and 30000 s^−1^, respectively (Fig. 4a). The pore-density at 30000 s^−1^ was lower than that at 25000 s^−1^ perhaps due to coalescence of the pores at high porosity.

The total number of carbon atoms as a function of time highlights the overall porosity of graphene. To understand the size and shape evolution of pores, the ratio of the number of edge atoms with that of the total atoms was probed (Fig. 5b). At the initial condition, this ratio was 0.03. As expected, this ratio changes under the CVD conditions in this study. At the etching rate constant of 17000 s^−1^, the ratio reduced close to zero, indicating that the graphene lattice became essentially nonporous. The ratio increased at higher etching rate constants (ratios of 0.077, 0.217, and 0.377 at k_et_ of 18000, 20000, and 25000 s^−1^, respectively). The ratio became close to half (0.45) at the etching rate constant of 30000 s^−1^. It is noteworthy that the ratio became constant after a period of time, indicating an equilibrium between the etching and the growth of graphene lattice at the pore edge. This is quite attractive for regulating the structure of the nanoporous graphene.

### Structure of graphene nanopores

**Figure 6:**
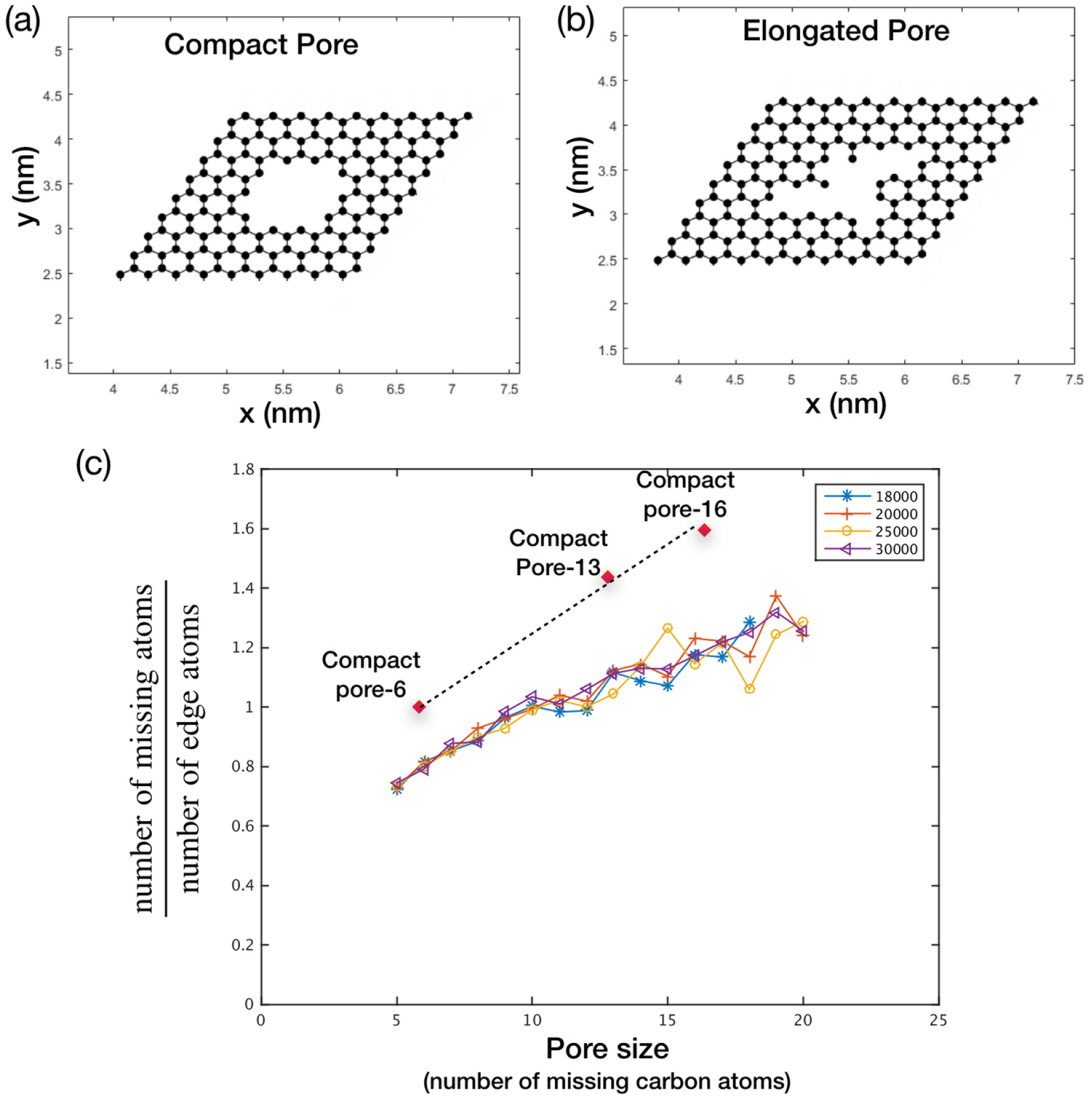
Representative compact (**a**) and elongated pores (**b**) with missing 16 carbon atoms. Structure of compact pore-6 and compact pore-13 is shown in Fig. S2. Here, pore-6 and pore-13 refer to compact pores made by missing 6 and 13 carbon atoms, respectively. (**c**) The ratio of the missing carbon atom vs. the edge atoms is plotted against the number of missing carbon atom. The figure highlights that most of the pores generated by the competitive etching in CVD condition are elongated.

The generated nanopores can be characterized by the number of missing carbon atoms as well as their shape. While, the number of missing carbon atoms may indicate nanopore area, for membrane application, it is not necessarily a true indicator of the edge-to-edge gap especially if the pores are elongated. This is important because even the pores with a large number of missing carbon atoms can be attractive for molecular sieving if they are elongated or slit-shaped. Therefore, to characterize the pore-shape, the ratio between the number of missing carbon atoms and the number of edge atoms was probed. Here, the edge atoms are defined by those carbon atoms that are connected by less than 3 carbon atoms. Generally, when the number of missing carbon atoms increases, the ratio tends to increase (Fig. 6). However, for a given number of missing carbon atoms, the ratio tends to be lower for elongated pores (Fig. 6b) mainly because elongated pores tend to have more edge atoms compared to the compact pores (Figs 6a and S2). Therefore, based on the ratio, one can judge whether a pore is compact or elongated. A comparison of this ratio for the pores generated with different etching rates indicates that the pores generated herein are somewhat elongated (Fig. 6c). This is advantageous for the membrane application because even if a substantial population of large pores was present in the nanoporous graphene lattice (for example, in the case of etching rate constants of 18000 s^−1^ and 20000 s^−1^), the lattice may still be attractive for the gas separation.

### Transport calculations

The gas transport across the nanoporous graphene was modeled adopting the adsorbed-phase transport model proposed by Strano and co-workers^20,21^. Briefly, the gas transport takes place in five key steps, (i) adsorption of the gas molecule on the surface of graphene on the feed side, (ii) diffusion of the molecule towards the pore and association with the pore on the feed side, (iii) translocation of the molecule through the pore from the feed side to the permeate side, (iv) dissociation of the molecule from the pore and subsequent diffusion away from the pore on the permeate side, and finally (v) desorption of the molecule from the graphene lattice on the permeate side. Typically, the rate-limiting step is the pore translocation step where the molecule arriving at a transition state in the center of the pore experiences an energy barrier from the electron cloud at the pore-edges. Therefore, the overall transport is activated with activation energy corresponding to the potential energy difference between the adsorbed state and the transition state. Our experimental measurements of gas transport through the intrinsic defects of graphene confirmed this, and a temperature activated gas transport was observed^18,19^. Accordingly, assuming the translocation step as the activated step and a rate controlling step, a mathematical model was derived to estimate the gas pair selectivity (section S1). Overall, the gas pair selectivity, *α*_*AB*_, from an ensemble of N pores, each with an area of *O*_*i*_ can be written as Eq. 1:

1 $${\alpha }_{AB}=\frac{{\sum }_{i=1}^{N}\,{O}_{i}\,\exp \,(-\frac{{\rm{\Delta }}{H}_{t.s.,A,i-gas,A}}{RT})\,}{{\sum }_{i=1}^{N}\,{O}_{i}\,\exp \,(-\frac{{\rm{\Delta }}{H}_{t.s.,B,i-gas,B}}{RT})}$$

where Δ*H*_*t*.*s*.,*A*,*i*−*gas*,*A*_ refers to the potential energy difference between two states of gas A when crossing pore *i*. These two states are transition-state of the gas during the pore-translocation when the molecule is in the cross-section of graphene pore, and the state in the gas phase. Similarly, Δ*H*_*t*.*s*.,*B*,*i*−*gas*,*B*_ refers to the potential energy difference between the corresponding two states of gas B. *T* is the temperature. *R* is the universal gas constant.

Calculating Δ*H*_*t*.*s*.,*A*,*i*−*gas*,*A*_ for more than a thousand pores generated in this study by traditional methods (ab-initio calculations or classical molecular dynamics simulations) is not feasible. Therefore, we developed a MATLAB based high-throughput algorithm probing the interaction of gases (He, H_2_, CO_2_, CH_4_ and N_2_) with the nanoporous graphene lattice and extracting the energy of the molecules at the transition state. Energy calculations were carried out considering the van der Waals interactions (6–12 Lennard-Jones or LJ potential, Eq. 2) and the electrostatic interactions (Eq. 3) between the gaseous molecules and the graphene nanopore.

2 $${E}_{LJ}=4\varepsilon \,[{(\frac{\sigma }{r})}^{12}-{(\frac{\sigma }{r})}^{6}]$$

3 $${E}_{electrostatic}=\frac{1}{4\pi {\epsilon }_{0}}\frac{{q}_{1}{q}_{2}}{r}$$

where, *r* is the distance between two atoms, *ε* and *σ* are the LJ parameters, *q*_*i*_ is the partial charge of atom *i*, and ${\epsilon }_{0}$ is the permittivity of vacuum.

The LJ interactions between different types of atoms were evaluated using the Lorentz-Berthelot mixing rule. A cut-off distance of 1.2 nm was applied to the LJ pairwise potentials. To be close to the realistic conditions of the pore-structure generated in the CVD process, the pore edges were hydrogen terminated. The parameters used in the simulation are listed in Table 2. The carbon atoms at the pore edge (connected with the H atoms) were treated differently from the interior carbon atoms (connected with three other C atoms). Carbon has a stronger electronegativity than hydrogen, and therefore, the edge carbon atoms possess negative partial charges and larger LJ parameters due to an increased electron density. The parameters for the edge carbon and the hydrogen atoms were calculated from those of benzene in the all-atom optimized potentials for liquid simulations (OPLS-AA) force field^32^. A constrained relaxation was carried out to study the potential energy surface for each molecule. The porous graphene sheet was held fixed, and the gas molecule was allowed to rotate freely to minimize the potential energy of the system. The potential energy calculation was carried out at 30 z-steps away from the basal plane of graphene (z = 0) with the step size of 0.02 nm. At each z-step, the position and the rotation of the gas molecule were optimized by calculating the minimum potential energy. The adsorption energy was assigned to the minimum potential energy along the z-steps. The transition-state (inside the nanopore) was assigned to the minimum potential energy at z equal to zero.

**Table 2:** The LJ parameters, bond lengths and partial charges used in our simulations to calculate the potential energy of gas molecules.

Lattice or molecule	Site	ε/k_B_	σ	q	Bond length (nm)	Reference
		(K)	(nm)	(e)		
Graphene	C	28.0	0.340	0	C-C (0.142)	^37^
	C (edge)	35.2	0.355	−0.115	C-H (0.109)	OPLS-AA
	H (edge)	15.1	0.242	0.115		
CO_2_	C	27.0	0.280	0.700	C=O (0.116)	^38^
	O	79.0	0.305	−0.350		
CH_4_	C	33.2	0.350	−0.240	C-H (0.109)	OPLS-AA
	H	15.1	0.250	0.060		
H_2_	H	0	0	0	H-H (0.074)	^39^
	center of mass	36.7	0.296	0		
He	He	10.2	0.258	0		^40^
N_2_	N center of mass	36.0 0	0.331 0	−0.482 0.964	N-N (0.110)	^41^

**Figure 7:**
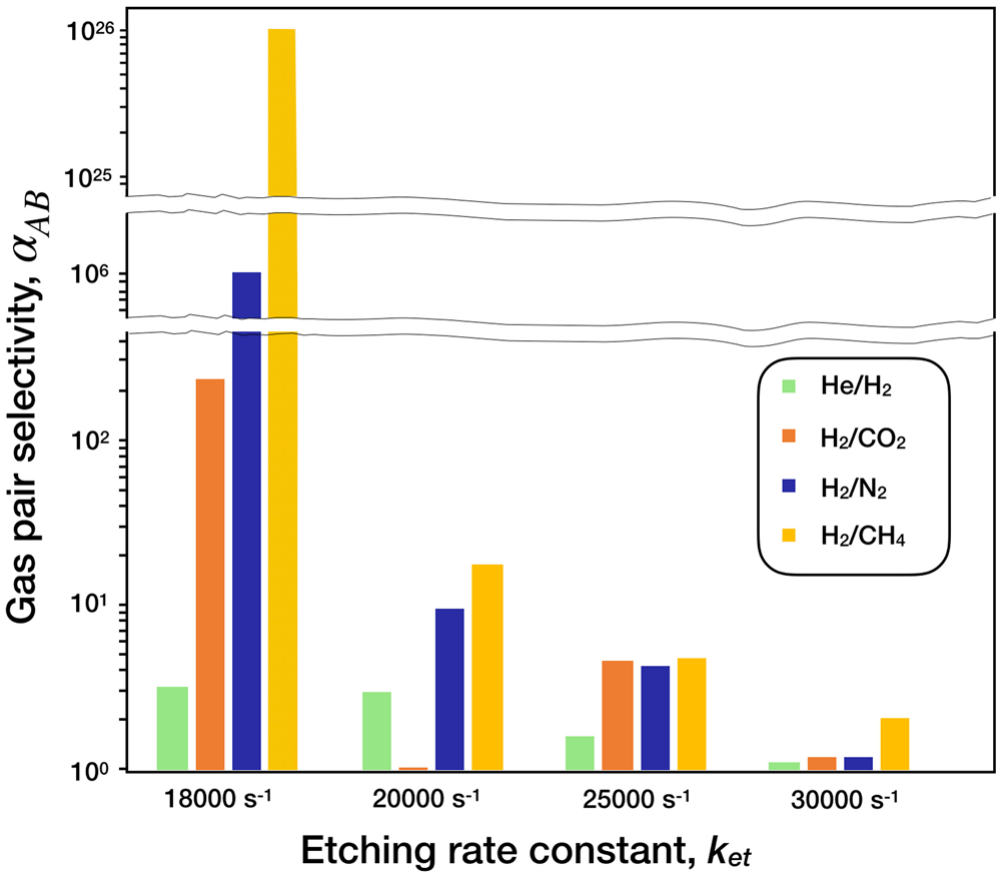
The estimated gas pair selectivity for various etching rate constants.

The model yielded adsorption energies of 1.9, 4.0, 8.5, 11.2 and 14.5 kJ/mole for He, H_2_, N_2,_ CH_4_ and CO_2_ respectively, on pristine graphene, which is in good agreement with the literature^33^. The corresponding adsorption heights of He, H_2_, N_2_, CH_4_ and CO_2_ on pristine graphene were 0.29, 0.31, 0.33, 0.35 and 0.43 nm, respectively. The ensemble of pores was simulated in the presence of these gas molecules to generate a dataset of Δ*H*_*t*.*s*.,*A*,*i*−*gas*,*A*_, from which the gas pair selectivity was calculated (Fig. 7 and Table S1). Attractive separation selectivities were obtained at the etching rate constants of 18000 s^−1^ and 20000 s^−1^. At 18000 s^−1^, He/H_2_, H_2_/CO_2_, H_2_/N_2_ and H_2_/CH_4_ selectivities were 3.2, 229, 10^6^ and 10^20^, respectively, indicating a sharp molecular cutoff between 0.29–0.33 nm, corresponding to the size gap between the kinetic diameters of H_2_ and CO_2_. Since the selectivities are consistent with molecular sieving effect where the molecule with the larger kinetic diameter has the lowest flux, the flux follows the trend of He > H_2_ > CO_2_ > N_2_ > CH_4_. The selectivity drops rapidly from the etching rate constant of 18000 s^−1^ to 20000 s^−1^, with H_2_/CH_4_ selectivity limited to 17.7. Increasing the etching rate constant beyond 20000 s^−1^ leads to a complete loss of selectivity, indicating the larger pores formed at higher etching rate constants act as defects, and dominate the overall transport. In general, the high gas selectivities in combination with the high pore-density (4.2 × 10^13^ cm^−2^) estimated here is highly attractive for the gas separation.

## Discussion

The development of nanoporous graphene hosting pore-density larger than 10^13^ cm^−2^ and sub-nanometer sized pores is extremely attractive for molecular separation. Our unique approach, involving the use of an etchant during the CVD of nanoporous graphene with CH_4_, leads to an equilibrium between the growth and etching of the edge atoms. As a result, beyond a critical CVD time, the total number of carbon atoms and edge atoms in the nanoporous graphene do not change as a function of time. Resulting nanoporous graphene structures show a high promise for gas separation with H_2_/CO_2_, H_2_/N_2_ and H_2_/CH_4_ separation selectivities greater than 200 and the pore-density greater than 10^13^ cm^−2^ at the etching rate constant of 18000 s^−1^. The realization of such membranes could be highly interesting for application in pre-combustion and post-combustion carbon capture, hydrogen purifications, and helium recovery from natural gas.

Overall, the competitive etching method introduced here overcomes the tradeoff between the mean pore-size and the pore-density typically observed with the conventional graphene etching methods (ion-bombardment, UV/ozone, plasma, etc.). The overall approach has a significant potential to be optimized with respect to etching rate constant, CH_4_ deposition rate, growth temperature, etc. The highly porous lattice of graphene is also expected to find applications in band-gap engineered optoelectronic devices.

## Method: Calculations

### kMC Simulation Method

In the CVD growth of graphene, four prominent events take place. These are: (a) adsorption of CH_4_ on the surface of Cu, (b) dehydrogenation of CH_4_ forming radicals, (c) surface diffusion of the radicals, and (d) the subsequent nucleation and growth of the graphene domains. When graphene lattice is already present on the Cu surface, the nucleation event is not required, and the growth precursors can directly attach to the existing lattice edges. Herein, we introduce an etchant in the CVD reactor along with the growth precursor (CH_4_) to tune the structure of nanoporous graphene (Fig. 1a). A mild etchant is chosen such that it only etches the existing graphene edges and such that it neither nucleates new defects on the basal plane nor etches the grain-boundaries. For instance, CO_2_ at 1000 °C satisfies these conditions, and only expands pre-existing intrinsic defects (Fig. S1). The purpose of such etchant in the CVD environment is to compete with the growth of graphene edges, ultimately tuning the pore topology based on the overall balance of reaction for growth and etching. The details of the model that is used to generate a porous structure are as follows:

### Adsorption of CH_4_

The physisorption of CH_4_ on Cu(111) surface was calculated according to the adsorption model where the deposition rate is dependent on the surface coverage (Eq. 4).

4 $${r}_{a}={r}_{a,0}[1-\theta (t)]$$

where *r*_*a*_ is the adsorption rate at time t, *r*_*a*,0_ is the initial adsorption rate, and *θ*(*t*) is the fractional surface coverage. In this simulation, CH_4_ can only be deposited on the fcc and hcp layer as mentioned in above section. Accordingly, there are 24960 sites for the deposition of CH_4_. It has been assumed that the gas molecules can be deposited into every site has equal probability and every deposition is successful (noted as CH_4_(s)). *r*_*a*_ can be modeled by the Knudsen’s formalism^34^, where the rate is dependent on the partial pressure of CH_4_ in the gas phase (noted as CH_4_(g)). As per Eq. 4, the deposition rate can be written as Eq. 5:

5 $${r}_{a}=PA[1-\theta (t)]/\sqrt{2\pi m{k}_{B}T}$$

where *P* is the CH_4_(g) partial pressure, and *m* is the molecular weight of CH_4_. T is the deposition temperature, and *k*_*B*_ is the Boltzmann constant, and *A* is the area of deposition.

### Dehydrogenation

After CH_4_(g) is adsorbed on the Cu(111) surface, Cu surface atoms catalyze the dehydrogenation reaction leading to the formation of growth precursors. The activation energies for the successive dehydrogenation steps (CH_4_(s) → CH_3_(s) → CH_2_(s) → CH(s) → C(s)) on Cu(111) have been calculated by Zhang *et al*. using DFT including the Arrhenius dependence, and are listed in Table 1 ^27^. The rate of the dehydrogenation step, ${r}_{d,C{H}_{i}(s)}$, for the CH_i_(s) species (where *i* = 1, 2, 3, 4) can be written as a first-order kinetics (Eq. 6).

6 $${r}_{d,C{H}_{i}(s)}={w}_{o}\,exp(-\frac{{E}_{act,d,C{H}_{i}(s)}}{{k}_{B}T}){N}_{C{H}_{i}(s)}$$

where ${N}_{C{H}_{i}(s)}$ represents the number of CH_i_(s) radicals on the surface, *w*_0_ is the frequency factor calculated from the Eyring equation and is equal to 2*k*_*B*_*T*/*h*, where *h* is the Plank’s constant. ${E}_{act,d,C{H}_{i}(s)}$ is the activation energy for the dehydrogenation of CH_i_(s). The overall rate of the process, *r*_*d*_, can be expressed as a summation of the dehydrogenation steps for all the species (Eq. 7).

7 $${r}_{d}=\sum _{i}\,{r}_{d,C{H}_{i}(s)}$$

For simplification, we have assumed that H_2_ is immediately removed from the surface (adsorbed inside the Cu lattice or released in the gas phase), and that it does not play a role in CVD. Consequently, the dehydrogenation step is irreversible and the concentrations of the intermediate radicals (CH_3_(s), CH_2_(s), CH(s)) are reduced. In this scenario, the surface diffusion of intermediate radicals can be neglected. Therefore, the crystallization of graphene proceeds by the surface diffusion of C(s), and subsequent bond formation.

### Surface diffusion

Hopping of carbon radicals from one adsorption site to another is marked as a single diffusion step. The single diffusion step is restricted to the nearest empty sites (sites that do not have carbon or other radicals). So, carbon on an hcp Cu site can jump to the nearest fcc sites and vice-varsa. Based on the number of carbon radicals close to the particular diffusing radical, the activation energy for diffusion will be different due to the interaction with the other carbon radicals. A large number of neighboring radicals would imply that the diffusing radical will need higher energy to arrive at the transient state. For example, Gaillard *et al*. calculated the surface diffusion energy considering the interaction with the neighbor (*n*_*ne*,*i*_) and the next to next neighbor (*n*_*nne*,*i*_) using the ab-initio calculations and expressed the activation energy for a particular type of diffusion as a function of *n*_*ne*,*i*_ and *n*_*nne*,*i*_^28^. In this case, for a carbon which is present in hcp site, it can have maximum three fcc neighbors and six next to next hcp neighbors and vice-varsa (Fig. 1b). The general form of activation energy for the surface diffusion of the *i*^*th*^ species, *E*_*act*,*sd*,*i*_, can be expressed as Eq. 8:

8 $${E}_{act,sd,i}={n}_{ne,i}{E}_{n}+{n}_{nne,i}{E}_{nn}$$

where *E*_*n*_ and *E*_*nn*_ are the activation energies for surface diffusion arising from interactions with the immediate neighbor and the next to the next neighbor, respectively. Accounting for the distribution in the activation energy, *E*_*act*,*sd*,*i*_, the overall rate of surface diffusion, *r*_*sd*_, can be written as Eq. 9:

9 $${r}_{sd}=\sum _{i=0}^{M}\,{v}_{i}{N}_{c(s),i}{w}_{o}\,exp(-\frac{{E}_{act,sd,i}}{{k}_{B}T})$$

where *N*_*C*(*s*),*i*_ is the number of carbon radicals associated with the activation energy of *E*_*act*,*sd*,*i*_. *v*_*i*_ is the number of directions the molecule can diffuse while taking into account the degree of freedom for the diffusion which is equal to the number of empty neighbor atom present.

### Etching

The etching is modeled with a first-order etching rate assuming a single etching rate constant. For example, CO_2_ can be used as an etchant which neither nucleates defects on graphene basal plane nor etches the grain boundaries (Fig. S1). The first-order etching rate can be expressed by Eq. 10:

10 $${r}_{et}={k}_{et}{N}_{C(s),edge}$$

where *k*_*et*_ is the first-order etching rate constant, and *N*_*C*(*s*),*edge*_ is the number of edge carbon atoms on the surface.

### kMC algorithm

The evolution of nanoporous graphene was simulated with the kMC algorithm to study system evolution at a large time-scale. The kMC algorithm was adopted from the study of Lucas *et. al*.^35^. Once a CH_4_(g) molecule is adsorbed on the Cu(111) surface, it gets dehydrogenated sequentially to form a carbon (C) radical. Next, the C radicals diffuse to the graphene nanopore edge and contribute to the edge growth. In parallel, nanopore edge atoms are etched by the etchant. According to this model, the deposition of CH_4_ on Cu occurs with a constant rate (Eq. 5). Between two successive CH_4_ deposition steps with time interval of Δ*t*_*a*_ (Eq. 11), surface events (dehydrogenation, surface diffusion, etching) can take place based on the respective rates (Eq. 12). In this fashion, the system evolves with time. The selection of an event at an instance is determined based on the relative rates of the events. The probability of an event with a higher rate will be higher (Eq. 13). If the probability among a number of species is equal, then the species for which the step is implemented is decided stochastically. With each event, the simulation time increases by Δ*t*_*s*_ (Eq. 12). After a deposition, when the summation of time intervals of surface events become larger than the time interval of deposition (Δ*t*_*s*_ > Δ*t*_*a*_), the next deposition of CH_4_ takes place, and again the Δ*t*_*s*_ count starts from zero.

11 $${\rm{\Delta }}{t}_{a}=\frac{1}{{r}_{a}}$$

12 $${\rm{\Delta }}{t}_{s}=\frac{1}{{r}_{d}+{r}_{sd}+{r}_{et}}$$

13 $${p}_{i}=\frac{{r}_{i}}{{r}_{d}+{r}_{sd}+{r}_{et}}$$

At first, the synthesis of pristine graphene domains on bare Cu(111) was demonstrated by introducing only CH_4_, validating the kMC model for the graphene growth (parameters for kMC simulation are shown in Table 1). Next, to simulate the evolution of nanoporous graphene, the Cu(111) surface was completely covered with the pristine graphene. Nanopores were created by stochastically removing carbon atoms from the lattice. This nanoporous graphene lattice served as a starting point for running the kMC simulation in the presence of CH_4_ as well as an etchant to probe the evolution of the PSD and the pore-density.

### Limitation of the kMC algorithm

Overall, the assumption made in the kMC algorithm are:

1. In our model, carbon atoms are only bound to the hcp and fcc layer of the copper lattice. This assumption follows from the calculation of Riikonen *et al*., where it was found that the sites on the Cu surface corresponding to the underlying hcp and the fcc cites are the most probable sites for the adsorption of carbon atoms^29^.
2. Because of the nature of the current setup, grain-boundary defects were not present in the graphene lattice. However, this does not affect the gas transport property of the graphene lattice because the grain-boundary defects are impermeable to gas molecules. Moreover, experimental evidence on etching of the polycrystalline graphene lattice by CO_2_ show that the grain-boundaries are resistant to etching (Fig. S1).
3. The current model assumes a single etching rate constant and ignores the differences in different edge configurations (armchair and zigzag). The zigzag edge has been shown to be more energetic than the armchair edge due to the confinement of P_z_ electrons on the edge atom. So, in principle, the etching of zigzag edge should be more probable than that of the armchair edge. However, at the crystallization temperature of 1000 °C, where the competitive growth and etching takes place, the edge reconstruction for energy minimization is expected to be significant, and it is highly likely that zigzag edges will relax to a low energy state such as zigzag57 which has similar energy as the armchair edge^36^, especially because the activation energy to do so is surmountable at 1000 °C. Therefore, the use of a single etching rate constant is justified at 1000 °C.

## Supplementary information

Supplementary Information

## Data Availability

The authors declare that all the data supporting the findings of this study are available within the article (and its Supplementary Information File), or available from the corresponding author on reasonable request.
