# Supplementary material for: Crystallization of gas-selective nanoporous graphene by competitive etching and growth: a modeling study
Source: Sci Rep. 2019 Mar 26;9:5202. doi: 10.1038/s41598-019-41645-9 (PMC6435714; doi:10.1038/s41598-019-41645-9)
Supplement: Supplementary file 1 — Supplementary Information [file 41598_2019_41645_MOESM1_ESM.pdf]

Crystallization of gas-selective nanoporous graphene by competitive etching and growth: a modeling study

Soumajit Dutta, Mohammad Tohidi Vahdat, Mojtaba Rezaei, Kumar Varoon Agrawal*

Laboratory of Advanced Separations (LAS), École Polytechnique Fédérale de Lausanne (EPFL), CH-1951 Sion, Switzerland.

E-mail: kumar.agrawal@epfl.ch

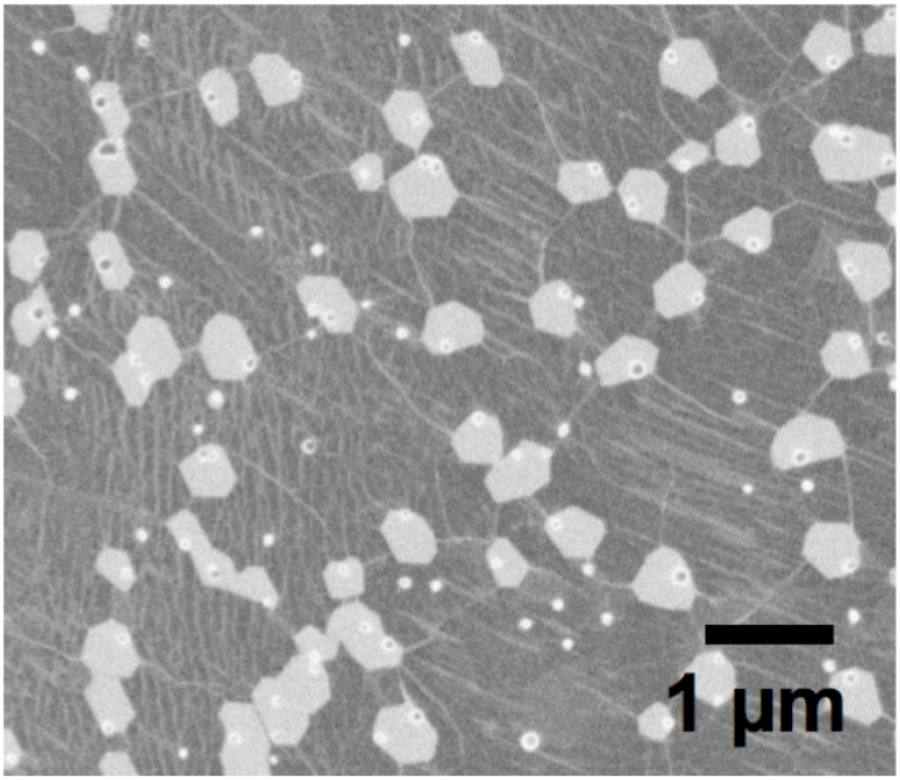

Figure S1: Etching of graphene grown by the chemical vapor deposition technique by CO_2 at 1000 ºC. Note that the grain boundaries remain intact and uniform. The pore-density is similar to that of the intrinsic defects.

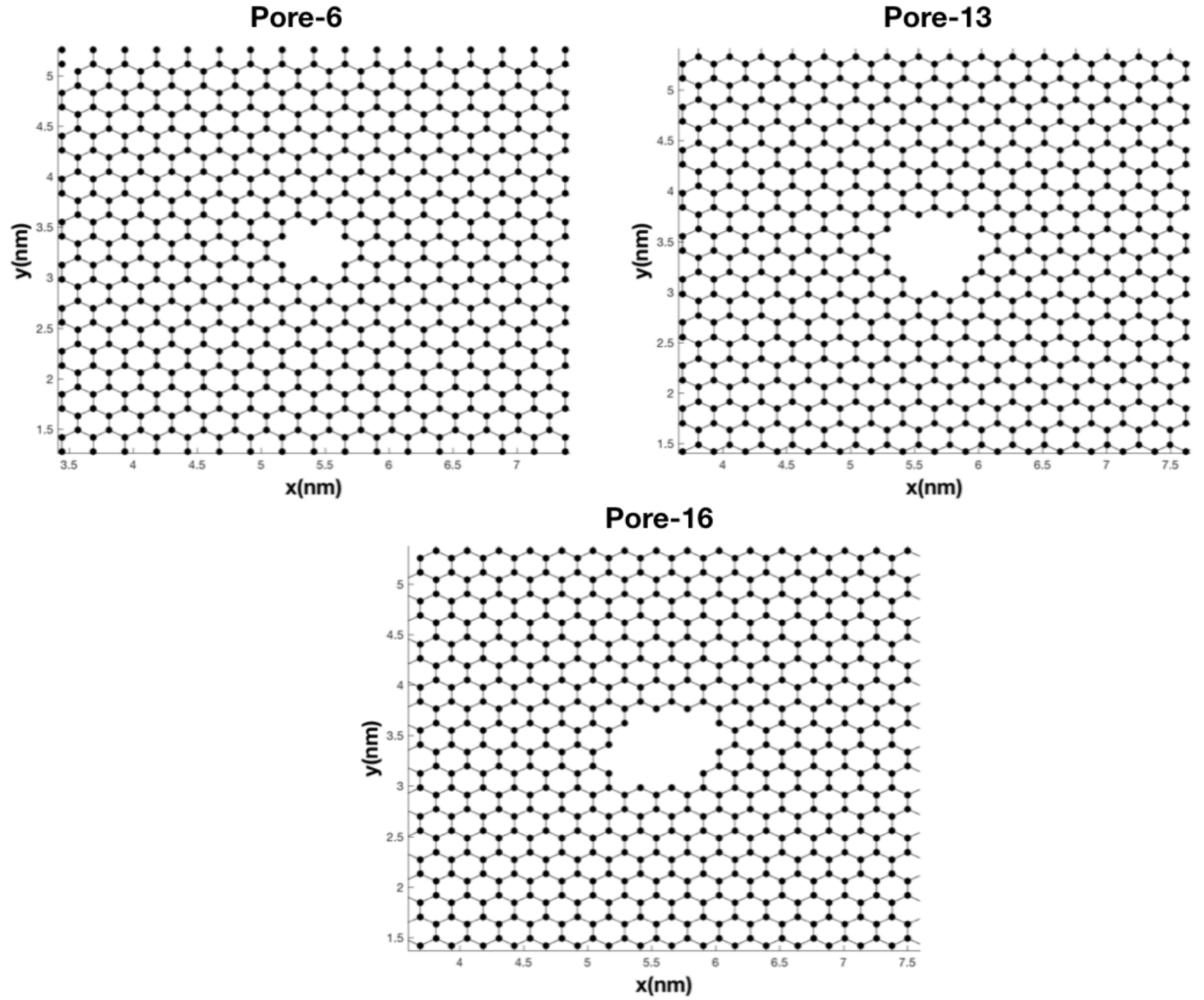

Figure S2: Representative compact pores made by missing 6, 13 and 16 carbon atoms (pore-6, pore-13 and pore-16, respectively).

Section S1: Transport Model

The adsorbed-phase transport of gases through nanoporous graphene can be summarized in 5 elementary steps as described by Drahushuk and Strano.[1] These steps include (Figure S3):

1) Adsorption of the gaseous molecule on the surface of graphene (feed side).

2) Two-dimensional diffusion of the molecule towards the pore and association with the pore (feed side).

3) Translocation through the pore.

4) Dissociation of the molecule from the pore, and diffusion of the molecule away from the pore (permeate side).

5) Desorption from the graphene lattice (permeate side).

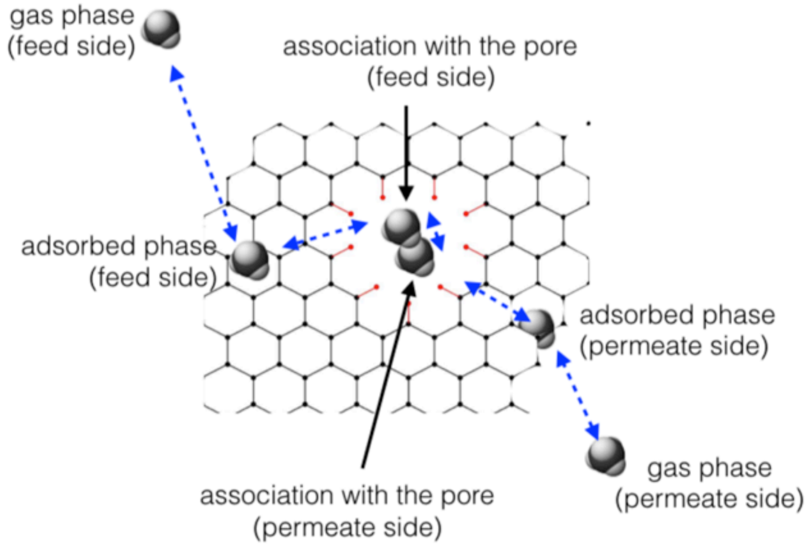

Figure S3: Schematic of the adsorbed-phase molecular transport through the nanoporous graphene.

These five steps can be described in the reaction kinetics notation. Below, we present a derivation of the molecular flux for the single-component feed:

Step 1 (adsorption):
$$F + l \underset{k_{-1}}{\overset{k_1}{\rightleftharpoons}} F_l \qquad \text{(S1)}$$

Step 2 (diffusion to pore):
$$F_l + O \underset{k_{-2}}{\overset{k_2}{\rightleftharpoons}} FO + l \qquad \text{(S2)}$$

Step 3 (pore translocation): $$FO \underset{k_{-3}}{\overset{k_3}{\rightleftharpoons}} PO' \tag{S3}$$

Step 4 (diffusion away from pore): $$PO' + l' \underset{k_{-4}}{\overset{k_4}{\rightleftharpoons}} P_{l'} + O' \tag{S4}$$

Step 5 (desorption): $$P_{l'} \underset{k_{-5}}{\overset{k_5}{\rightleftharpoons}} l' + P \tag{S5}$$

where, F = Gaseous species on the feed side with feed pressure P_F

l = Concentration of the available graphene lattice sites for adsorption (feed side)

F_l = Concentration of the molecule on the graphene lattice (feed side)

O = Concentration of the empty pores (feed side)

FO = Concentration of the pores associated with the molecule (feed side)

PO' = Concentration of the pores associated with the molecule (permeate side)

l' = Concentration of the available graphene lattice sites for adsorption (permeate side)

$P_{l'}$ = Concentration of the molecule on the graphene lattice (permeate side)

P = Gaseous species on the permeate side with permeate pressure P_P

k_i = forward rate constant for step i

k_{-i} = backward rate constant for step i

Typically, the rate-limiting step is the translocation step (step 3), where the molecule associated with a pore experiences an activation barrier to translocate the pore. The other four steps can be considered to be at equilibrium. Therefore,

$$Equilibrium\ adsorption:\ K_1 = \frac{k_1}{k_{-1}} = \frac{[F_l]}{P_F[l]} \tag{S6}$$

$$Equilibrium\ \ diffusion: K_2 = \frac{k_2}{k_{-2}} = \frac{[FO][l]}{[F_l][O]} = \frac{[FO]}{[O]P_F K_1} \tag{S7}$$

$$Equilibrium\ \ diffusion: K_4 = \frac{k_4}{k_{-4}} = \frac{[P_{l'}][O']}{[l'][PO']} \tag{S8}$$

$$Equilibrium\ desorption: K_5 = \frac{k_5}{k_{-5}} = \frac{P_P[l']}{[P_{l'}]} \tag{S9}$$

Also, the total adsorption site, C_l, is fixed such that:

$$C_l = [F_l] + [l] = [P_{l'}] + [l'] \qquad \text{(S10)}$$

Similarly, the total number of pores, C_o, is fixed such that:

$$C_o = [O] + [FO] = [O'] + [PO'] \qquad \text{(S11)}$$

The Langmuir adsorption model can describe the adsorption of gases on the graphene lattice, especially at mild pressures (i.e., 1.6 bars used in the experiment) and temperature above 300K. Therefore,

$$\theta_F = fraction\ site\ occupancy = \frac{[F_l]}{C_l} = \frac{K_1 P_F}{1+K_1 P_F} \qquad \text{(S12)}$$

Combining Equations S6, S7, S10, S11, and S12 yields:

$$[F_l] = \frac{C_l K_1 P_F}{1+K_1 P_F} \qquad \text{(S13)}$$

$$[l] = \frac{C_l}{1+K_1 P_F} \qquad \text{(S14)}$$

$$[FO] = \frac{C_o K_1 K_2 P_F}{1+K_1 K_2 P_F} \qquad \text{(S15)}$$

Since the structure and properties of the graphene membrane can be assumed to be symmetric on both sides of the graphene sheet, one can write:

$$K_4 = 1/K_2 \qquad \text{(S16)}$$

$$K_5 = 1/K_1 \qquad \text{(S17)}$$

$$K_3 = K_{-3} \qquad \text{(S18)}$$

Similarly, combining Equations S8, S9, S10, S11, S16, and S17, and assuming Langmuir desorption model, yields:

$$[PO'] = \frac{C_o K_1 K_2 P_P}{1+K_1 K_2 P_P} \qquad \text{(S19)}$$

Overall gas flux = rate of molecular translocation through the pores = $k_3[FO] - k_{-3}[PO']$

Therefore, overall flux = $k_3([FO] - [PO']) = C_o K_1 K_2 k_3 \left(\frac{P_F}{1+K_1 K_2 P_F} - \frac{P_P}{1+K_1 K_2 P_P}\right)$ (S20)

Flux per pore can be obtained by dividing Equation S20 by C_o

Flux per pore = $K_1K_2k_3\left(\frac{P_F}{1+K_1K_2P_F} - \frac{P_P}{1+K_1K_2P_P}\right)$ (S21)

Classical transition-state theory (CTST)[2] indicates that:

$$k_3 = A_{act}exp\left(-\frac{E_{act}}{RT}\right) \quad \text{(S22)}$$

where E_{act} is the activation energy for pore translocation, and A_{act} is the pre-exponential coefficient for translocation. Using the Van't Hoff's formalism, K_1 and K_2 can be obtained by Equations S23 and S24.

$$K_1 = exp\left(\frac{\Delta S_{\mathrm{ads}}}{R}\right) exp\left(-\frac{\Delta H_{\mathrm{ads}}}{RT}\right) \quad \text{(S23)}$$

$$K_2 = exp\left(\frac{\Delta S_{\mathrm{2D}}}{R}\right) exp\left(-\frac{\Delta H_{\mathrm{2D}}}{RT}\right) \quad \text{(S24)}$$

where ΔS_{ads} and ΔH_{ads} are changes in the entropy and the enthalpy of the system, respectively, upon gas adsorption to the pristine graphene lattice. Similarly, ΔS_{2D} and ΔH_{2D} are changes in the entropy and the enthalpy of the system, respectively, upon association of the gas to the graphene nanopore. Therefore, Equation S21 can be rewritten as follows:

Gas flux per pore = $A_{act}\ exp\left(\frac{\Delta S_{\mathrm{ads}}+\Delta S_{\mathrm{2D}}}{R}\right) exp\left(-\frac{(E_{act}+\Delta H_{\mathrm{ads}}+\Delta H_{\mathrm{2D}})}{RT}\right)\left(\mathrm{f}(P_f)-\mathrm{f}(P_p)\right)$ (S26)

where, $\mathrm{f}(P_f) = \frac{P_f}{1+exp\left(\frac{\Delta S_{\mathrm{ads}}+\Delta S_{\mathrm{2D}}}{R}\right) exp\left(-\frac{\Delta H_{\mathrm{ads}}+\Delta H_{\mathrm{2D}}}{RT}\right)P_f}$

and $\mathrm{f}(P_p) = \frac{P_p}{1+exp\left(\frac{\Delta S_{\mathrm{ads}}+\Delta S_{\mathrm{2D}}}{R}\right) exp\left(-\frac{\Delta H_{\mathrm{ads}}+\Delta H_{\mathrm{2D}}}{RT}\right)P_p}$

Approximations:

For light gases such as H_2, He, CO_2, N_2, and CH_4, the adsorption on graphene is not extremely strong, and therefore,

$$\mathrm{f}(P_f) = P_f \quad \text{(S27)}$$

$$\mathrm{f}(P_p) = P_p \quad \text{(S28)}$$

Therefore, gas flux per pore = $A_{act}\ exp\left(\frac{\Delta S_{\mathrm{ads}}+\Delta S_{\mathrm{2D}}}{R}\right) exp\left(-\frac{(E_{act}+\Delta H_{\mathrm{ads}}+\Delta H_{\mathrm{2D}})}{RT}\right)(P_f - P_p)$ (S29)

E_{act}, ΔH_{ads} and ΔH_{2D} can be described by Equations S30-32

$$E_{\text{act}} = H_{t.s.} - H_{pore\_ads} \tag{S30}$$

$$\Delta H_{\text{ads}} = H_{graphene\_ads} - H_{gas} \tag{S31}$$

$$\Delta H_{\text{2D}} = H_{pore\_ads} - H_{graphene\_ads} \tag{S32}$$

where $H_{t.s.}$ and H_{pore_ads} are the enthalpy of the gas at the transition state during the pore translocation step and at the physisorbed state on the pore, respectively. H_{gas} and $H_{graphene\_ads}$ are the enthalpy of the gas molecule in the gas phase and in the adsorbed state on the graphene lattice, respectively. Therefore,

$$E_{\text{act}} + \Delta H_{\text{ads}} + \Delta H_{\text{2D}} = H_{t.s.} - H_{gas} = \Delta H_{t.s.-gas} \tag{S33}$$

Similarly,

$$\Delta S_{ads} + \Delta S_{\text{2D}} = S_{pore\_ads} - S_{gas} = \Delta S_{pore\_ads-gas} \tag{S34}$$

where $S_{pore-ads}$ and S_{gas} are the entropy of the gas molecule in the adsorbed state on the pore and in the gas phase, respectively.

Therefore, gas flux per pore = $A_{act}\, exp\left(\frac{\Delta S_{pore\_ads-gas}}{R}\right) exp\left(-\frac{\Delta H_{t.s.-gas}}{RT}\right) \left(P_f - P_p\right)$ (S35)

For gases A and B, the selectivity, α_{AB}, from a given pore is calculated by dividing the flux for gas B from the flux of gas A. Generally, A_{act} for gases are similar (10^{13} s^{-1}). Also, the relative changes in entropy is much smaller than the relative changes in enthalpy, and therefore from a system of N pores, each with an open area O_i, the gas selectivity can be approximated by Equation S36

$$\alpha_{AB} = ratio\ of\ total\ flow\ rates = \frac{\sum_{i=1}^{N} O_i * Flux_A}{\sum_{i=1}^{N} O_i * Flux_B} = \frac{\sum_{i=1}^{N} O_i * exp\left(-\frac{\Delta H_{t.s.,A,i-gas,A}}{RT}\right)}{\sum_{i=1}^{N} O_i * exp\left(-\frac{\Delta H_{t.s.,B,i-gas,B}}{RT}\right)} \tag{S36}$$

Table S1. Gas pair selectivity

k_{et}	He/H_2	H_2/CO_2	H_2/N_2	H_2/CH_4
18000 s^{-1}	3.2	229	1.0E+06	1.0E+20
20000 s^{-1}	2.9	1.0	9.5	17.7
25000 s^{-1}	1.6	4.5	4.2	4.8
30000 s^{-1}	1.1	1.2	1.2	2.0

References

1. Drahushuk, L. W. & Strano, M. S. Mechanisms of gas permeation through single layer graphene membranes. *Langmuir* **28,** 16671–8 (2012).

2. Kolasinski, K. W. in *Surface Science: Foundations of Catalysis and Nanoscience* (2012).

3. Cheng, A. & Steele, W. a. Computer simulation of ammonia on graphite. I. Low temperature structure of monolayer and bilayer films. *J. Chem. Phys.* **92,** 3858 (1990).

4. Potoff, J. J. & Siepmann, J. I. Vapor–liquid equilibria of mixtures containing alkanes, carbon dioxide, and nitrogen. *AIChE J.* **47,** 1676–1682 (2001).

5. Yang, Q. & Zhong, C. Molecular Simulation of Carbon Dioxide/Methane/Hydrogen Mixture Adsorption in Metal−Organic Frameworks. *J. Phys. Chem. B* **110,** 17776–17783 (2006).

6. Sok, R. M., Berendsen, H. J. C. & van Gunsteren, W. F. Molecular dynamics simulation of the transport of small molecules across a polymer membrane. *J. Chem. Phys.* **96,** 4699–4704 (1992).

7. Karra, J. R. & Walton, K. S. Effect of Open Metal Sites on Adsorption of Polar and Nonpolar Molecules in Metal−Organic Framework Cu-BTC. *Langmuir* **24,** 8620–8626 (2008).
